# Post-migration stress mediates associations between potentially traumatic peri-migration experiences and mental health among Middle Eastern refugees in Germany

**DOI:** 10.1186/s12889-025-23660-w

**Published:** 2025-07-29

**Authors:** Usama EL-Awad, Robert Eves, Justin Hachenberger, Kayvan Bozorgmehr, Theresa M. Entringer, Tobias Hecker, Oliver Razum, Odile Sauzet, Sakari Lemola

**Affiliations:** 1 Faculty of Psychology and Sports Sciences, Bielefeld University, P.O. Box 10 01 31, Bielefeld, 33501 Germany; 2 Faculty of Medicine, University Clinic of Psychiatry and Psychotherapy, Virchowstraße 65, 32312 Luebbecke, Ruhr University Bochum (Campus OWL), Bochum, Germany; 3 School of Public Health, Department of Population Medicine and Health Services Research, Bielefeld University, P.O. Box 10 01 31, Bielefeld, 33501 Germany; 4 Section Health Equity Studies & Migration, Heidelberg University Hospital, Heidelberg, Germany; 5 Institute for Interdisciplinary Research on Conflict & Violence, Bielefeld University, P.O. Box 10 01 31, Bielefeld, 33501 Germany; 6 Institute of Psychology, University of Greifswald, Greifswald, Germany; 7 German Socio-Economic Panel, German Institute for Economic Research (DIW Berlin), Berlin, Germany; 8 School of Public Health, Department of Epidemiology and International Public Health, Bielefeld University, P.O. Box 10 01 31, Bielefeld, 33501 Germany; 9 Department of Business Administration and Economics, Bielefeld University, P.O. Box 10 01 31, Bielefeld, 33501 Germany

**Keywords:** Peri-migration stressors, Post-migration stress, Psychological distress, Causal mediation, Refugee mental health, Discrimination

## Abstract

**Background:**

On their way to host countries, refugees are often exposed to severe adversity, including cumulative experiences of fraud, extortion, robbery, detention, and shipwrecks, as well as prolonged, life-threatening small boat crossings. However, little research has examined the long-term impact of such peri-migration stressors on subsequent stress and mental health after arrival. This study explored how cumulative exposure to potentially traumatic events (PTEs) and small boat crossings before arrival affected psychological distress in Middle Eastern refugees, considering the mediating role of post-migration stress in the years following resettlement in Germany.

**Methods:**

Longitudinal data from the IAB-BAMF-SOEP Survey of Refugees collected at three survey waves (2016, 2018, and 2020; *N* = 541, *M*_age_ = 38.77, 14% female) were analyzed using a causal mediation approach with linear mixed models. Self-reports of cumulative exposure to PTEs and small boat crossings in the Mediterranean indicated peri-migration stressors, while perceived discrimination and migration-related worries reflected post-migration stress. Psychological distress was assessed using a general health questionnaire.

**Results:**

PTEs were significantly associated with higher migration-related worries (β = 0.10, *p* < .001). Migration-related worries significantly mediated the association between PTEs and psychological distress (ACME = 0.02, 95% CI [0.01, 0.04], *p* < .001; ADE = 0.07, 95% CI [0.03, 0.11], *p* < .001). In contrast, perceived discrimination did not mediate this link. However, small boat crossings were significantly associated with higher perceived discrimination (β = 0.11, *p* < .001), which in turn mediated their association with psychological distress (ACME = 0.05, 95% CI [0.02, 0.08], *p* < .001), while the direct effect was non-significant. These indirect effects intensified over time.

**Conclusions:**

Peri-migration stressors may contribute to long-term mental health issues in refugees via their post-migration stress experiences. However, these mediation pathways may differ, underscoring the need for nuance and further investigation. Addressing both early traumatic experiences and post-migration adversities, such as discrimination and migration-related worries, is crucial to mitigating refugees’ long-term psychological distress. These findings highlight the importance of early prevention and intervention efforts that address post-migration stress as a key factor in reducing the long-term mental health burden among refugees.

**Supplementary Information:**

The online version contains supplementary material available at 10.1186/s12889-025-23660-w.

## Introduction

The global displacement crisis continues to grow, with the number of refugees under UNHCR’s mandate nearly doubling since 2015, alongside a more than twofold increase in asylum seekers [1]. In Germany, over 1 million refugees from the Middle East, predominantly from Syria, Afghanistan, and Iraq, have sought refuge in the past decade due to wars and violent conflicts in the region [2].

### Stress related to migration before arrival

Refugees are constantly exposed to stressors before, during, and after their migration that can affect their mental health. Depending on the time of exposure, literature often distinguishes between different phases of perceived migration-related stress: *Pre-migration stress* involves responses to adverse events in the country of origin, such as political instability, violence, poverty, chronic illness, and limited access to healthcare services [3, 4]. Due to the nature of forced migration, refugees are frequently exposed to multiple, cumulative *potentially traumatic events* (PTEs), both before leaving their home country and along the migration route. A substantial body of research has demonstrated that such experiences can have enduring effects on mental health well beyond the point of resettlement [5–8]. In line with the dose–response principle, evidence further suggests that exposure to a greater number of distinct PTEs is associated with increasingly adverse health outcomes [7]. Events occurring during the forced migration are typically referred to as *peri-migration stressors* and include physical and sexual assault, witnessing violence, suffering a shipwreck and detention by enemy soldiers or police, for example [9]. Furthermore, a specific peri-migration stressor for many refugees from the Middle East and Africa is the crossing of the Mediterranean Sea in small, overcrowded, and often unseaworthy boats—typically undertaken without legal protection or adequate safety measures. *Small boat crossings* should not be understood as acute episodes of violence or coercion, but rather as risk environments shaped by systemic constraints and migratory conditions. Since the closure of the Balkan route in 2016, small boat crossings have constituted a key escape route to Europe for many refugees, with such passages often taking from several days to several weeks [10–12]. Given their inherent features of prolonged uncertainty, limited physical safety, helplessness, lack of control over the course of the situation, and complete dependence on third parties including smugglers, these crossings can be considered as significant precarious peri-migration stressors [13, 14]. Such conditions are typically characterized by collective tension, heightened psychophysiological stress, and an ongoing sense of vulnerability [15]. For instance, Tofani et al. [16] found that refugees who reported having crossed the Mediterranean Sea via small boats were significantly more likely to report anxiety-related impairments than those who used alternative migration route. Moreover, under particularly hazardous conditions, small boat crossings may escalate into acute, life-threatening experiences that could meet the criteria for PTEs. Specific events such as adverse weather, near-drownings, witnessing death at sea or forced returns (push-backs) illustrate how a structurally high-risk mode of mobility can evolve into a direct source of trauma [15, 17]. In this sense, small boat crossings represent both a sustained form of psychosocial stress and a situational context for the emergence of PTEs.

### Post-migration stress and its implications for refugee mental health

We conceptualize post-migration stress as the subjective experience of psychosocial burden arising from conditions encountered after resettlement. This includes both subjectively assessed stressors—such as perceived discrimination—and psychological manifestations—such as persistent migration-related worries—that reflect the ongoing mental strain associated with adapting to life in the host country. Such stress may be exacerbated in response to, for instance, language barriers, legal insecurity, financial hardship, cultural dissonance, discrimination, and inadequate living conditions [18–25]. Although the nature and quality of these conditions may vary depending on the region of origin and place of residence, they are often considered particularly burdensome for refugees from the Middle East in Europe—likely because of pronounced cultural and religious differences that may increase the risk of social exclusion and discrimination including structural barriers and public narratives that may reinforce stigmatization and perceived rejection, as well as the existential uncertainty associated with an insecure legal status [26–28]. This study therefore focuses on two specific forms of post-migration stress that are described in the literature as being particularly closely linked to mental health outcomes among Middle Eastern refugees: (1) *Migration-related worries* refer to persistent chronic uncertainties in everyday life, such as the outcome of the asylum procedure, financial security, health, or residence status, which are processed cognitively and emotionally (e.g [29, 30]). (2) *Perceived discrimination*, in turn, describes the experience of social rejection or disadvantage based on one’s origin, religion, or membership in a refugee group—a central feature of cultural stress among migrants and refugees [31, 32]. However, it remains unclear how the experience of pre-, peri- and post-migration stress are interrelated and may influence mental health outcomes of refugees such as psychological distress. A central and largely unresolved question is whether the effects of peri-migration stressors—such as cumulatively experienced PTEs or specific stressors such as small boat crossing—have a direct effect on mental health or whether their effect is mediated by post-migration stress responses, such as experiences of discrimination and migration-related worries in daily life, which may also vary depending on the type of stressor or stress, age, and gender.

### Applying the Stress Proliferation Theory to the refugee experience

To conceptually frame the relationship between early and later stress exposures and their potential impact on the mental health of refugees during the initial period of resettlement in the host country, the present study draws on Stress Proliferation Theory (SPT) [33]. The notion that post-migration stress can at least partially mediate the long-term mental health effects of peri-migration stressors aligns with the conceptual framework of this approach that distinguishes between stressful events and their consequences depending on the time of occurrence and quality. Primary stressors are understood as initial stress-inducing events or conditions that—due to their duration, uncontrollability, or relevance to core life domains—may initiate a cascade of further, secondary stressors over time [33]. Applied to the refugee context, PTEs and small boat crossings can be classified as primary stressors, while challenges that arise later in the host country and may lead to migration-related worries and perceptions of discrimination can be classified as subjective psychological strain resulting from secondary stressors [30, 34–36]. It can be assumed that individuals who encountered cumulative or particularly demanding peri-migration stressors may be more likely to face exposure to post-migration stress due to downstream social and structural consequences of trauma—such as unstable living conditions, lower access to resources, or disrupted support networks [37, 38]. Additionally, trauma-related cognitive and emotional patterns may trigger if and how subsequent challenges are perceived and experienced [39]. Furthermore, the exposure of earlier migration-related stressors can also have a main effect on mental health outcomes, independently of the indirect effect of perceived post-migration stress as research could indicate: Li & Anderson [35], for example, showed that traumatic experiences prior to arrival were associated with psychological distress among Asian American immigrants, both directly and indirectly through perceived discrimination. Goodkind et al. [34] identified post-migration stress in financial context as a mediating factor between PTEs and mental health outcomes including PTSD symptoms. Similarly, Yilmaz et al. [30] found mediation through worries about cultural identity among Syrian refugees in Turkey. Finally, Lohaus et al. [40] demonstrated that culturally influenced post-migration stress partially explains the association between PTEs and psychological distress among young refugees from the Middle East in Germany. Some studies, however, have reported exclusively indirect effects of PTEs on mental health via post-migration stress including worries and perceived discrimination—such as a study among young Middle Eastern refugees in Norway [29].

### The present study

While some studies focus primarily on pre-migration stressors or do not clearly distinguish between pre- and peri-migration stress, the present study explicitly addresses the gap in the literature by specifically examining refugees’ exposure to peri-migration stressors. Particularly relevant stressors relate to the cumulative experiences of PTEs and context-specific experiences such as small boat crossings in the Mediterranean among Middle Eastern refugees. Developing targeted assessment instruments and intervention strategies requires a better understanding of how the exposure to peri-migration stressors relate to later experiences of post-migration stress—such as migration-related worries or perceived discrimination—and how these, in turn, may affect the mental health of refugees.

We focus specifically on Middle Eastern refugees from Syria, Afghanistan, and Iraq who arrived between 2013 and 2016 in Germany and were surveyed for the first time in 2016 (with a maximum length of stay of up to three years), as this group represents a significant proportion of forced migrants due to wars and conflicts during this period. While previous studies primarily rely on cross-sectional analyses that offer only a snapshot of the hypothesized relationships, our study utilizes a longitudinal framework. To the best of the authors’ knowledge, there has been no longitudinal study so far that examines the relationship between the experience of peri-migration stressors and post-migration stress in relation to mental health outcomes in a group of Middle Eastern refugees who are living in Europe since arriving a few years ago. Our analysis is based on the longitudinal data from the German IAB-BAMF-SOEP Survey on Refugees [41, 42], conducted by the German Institute for Employment Research (IAB), the German Federal Office for Migration and Refugees (BAMF), and the German Socio-Economic Panel (SOEP). By applying a causal mediation approach using linear mixed models, the analysis incorporates data from three survey waves, enabling the capture of variations in stress and mental health outcomes across time and offering a comprehensive longitudinal perspective.

**Fig. 1:**
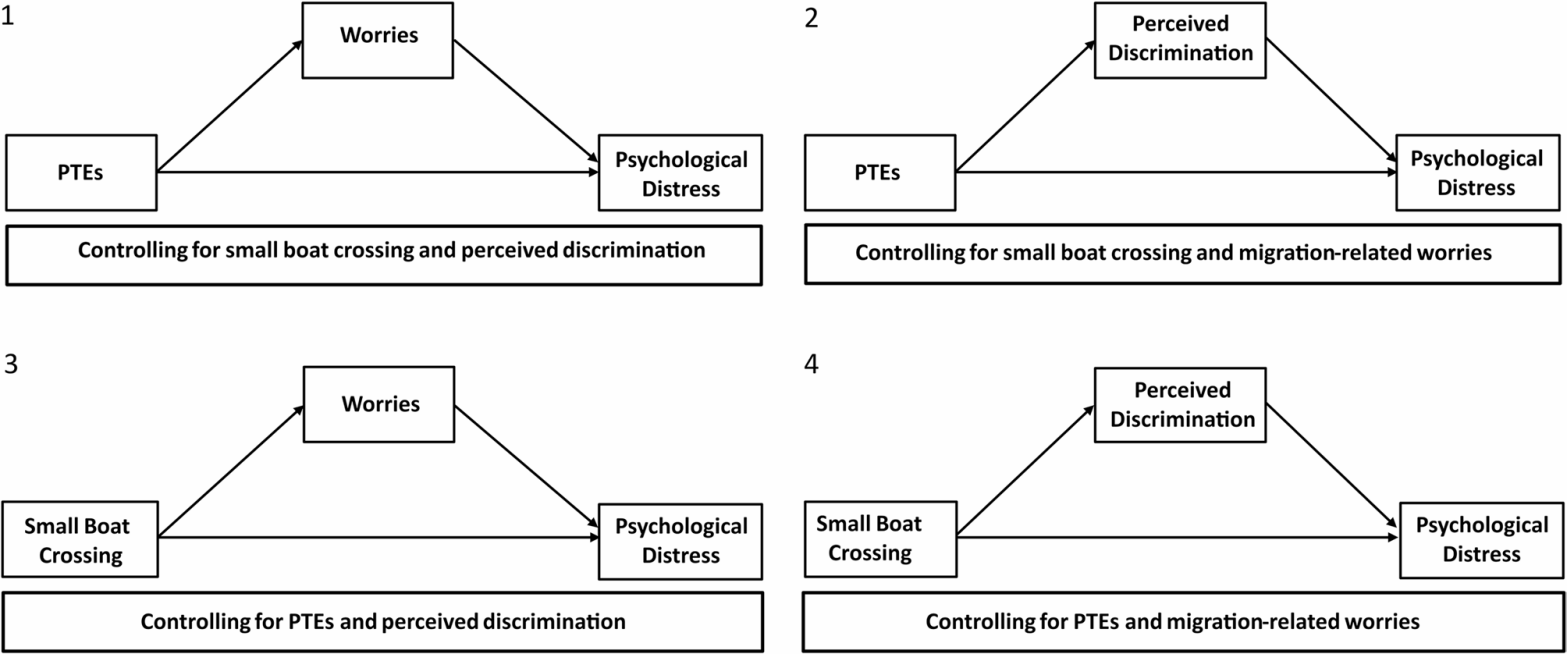
Migration-related worries and perceived discrimination as mediators between PTEs and psychological distress. Pre-migration stressors (time-invariant):PTEs = potentially traumatic events experienced during migration; Small boat crossing = the use of the Mediterranean route by boat during migration. Post-migration stress (time-variant): Worries = Having concerns in various migration-related areas of everyday life. Mental Health (time-variant): Psychological distress. All models control for (1) age, (2) gender, (3) subjective assessment of relative net income before migration (time-invariant), (4) current housing situation (time-variant), (5) German language proficiency (time-variant), and (6) whether there was support from relatives when moving to Germany (time-invariant)

In line with the SPT, the primary objectives and hypotheses of this study are:

1. *To examine whether peri-migration stressors predict post-migration stress.* We expect that a greater cumulative exposure of potentially traumatic experiences or a small boat crossing during migration as a specific potentially traumatic event will significantly predict greater perceived discrimination and migration-related worries across three annual points extending over approximately six years.
2. *To assess whether post-migration stress mediates the impact of peri-migration stressors on mental health*. We hypothesize that migration-related worries and perceived discrimination mediate the relationship between a greater cumulative exposure of potentially traumatic experiences or a small boat crossing during migration and psychological distress after resettlement (see Fig. 1).

We expect these effects to be robust despite adjusting several time-invariant and time-variant control variables, including (1) age, (2) gender, (3) subjective assessment of relative income before migration, as a proxy for pre- and peri-migration socioeconomic resources, (4) current housing situation, (5) German language proficiency, and (6) whether there was support from relatives when moving to Germany, which may buffer stress exposure through social support.

## Method

### Data and ethical statement

The data used for this study comes from the IAB-BAMF-SOEP Survey of Refugees [42] and are accessible to scientists and included in the SOEP-Core v38 data [41]. The dataset is anonymized and complies with all relevant data protection regulations. As a joint project of the German Institute for Employment Research (IAB), the Research Center of the German Federal Office for Migration and Refugees (BAMF-FZ) and the German Socio-Economic Panel (SOEP), the first part of the sample (M3) was financed by IAB research funds, while the second part (M4) was financed by funds from the BMBF and focused on refugee families. The survey was conducted in accordance with the applicable ethical standards. A stratified, clustered sample was drawn from the Central Register of Foreigners to represent refugees arriving between January 2013 and June 2016 [42], who were contacted in advance by mail. Informed consent was obtained from all participants, before they were asked to answer questions on education, training, health and integration in annual computer-assisted personal interviews, partly with the assistance of language mediators. All interview materials (letters, flyers, questionnaires) were translated by professional translators into seven languages (Arabic, Farsi, Kurmanji, Pashto, Urdu, English, and German), using a double translation and harmonization process [43]. During the interviews, both German and the language preferred by the interviewee were displayed on the screen at the same time. The first wave of data collection took place in 2016, meaning that participants had resided in Germany for up to three years at that time.

### Sampling strategy

**Fig. 2:**
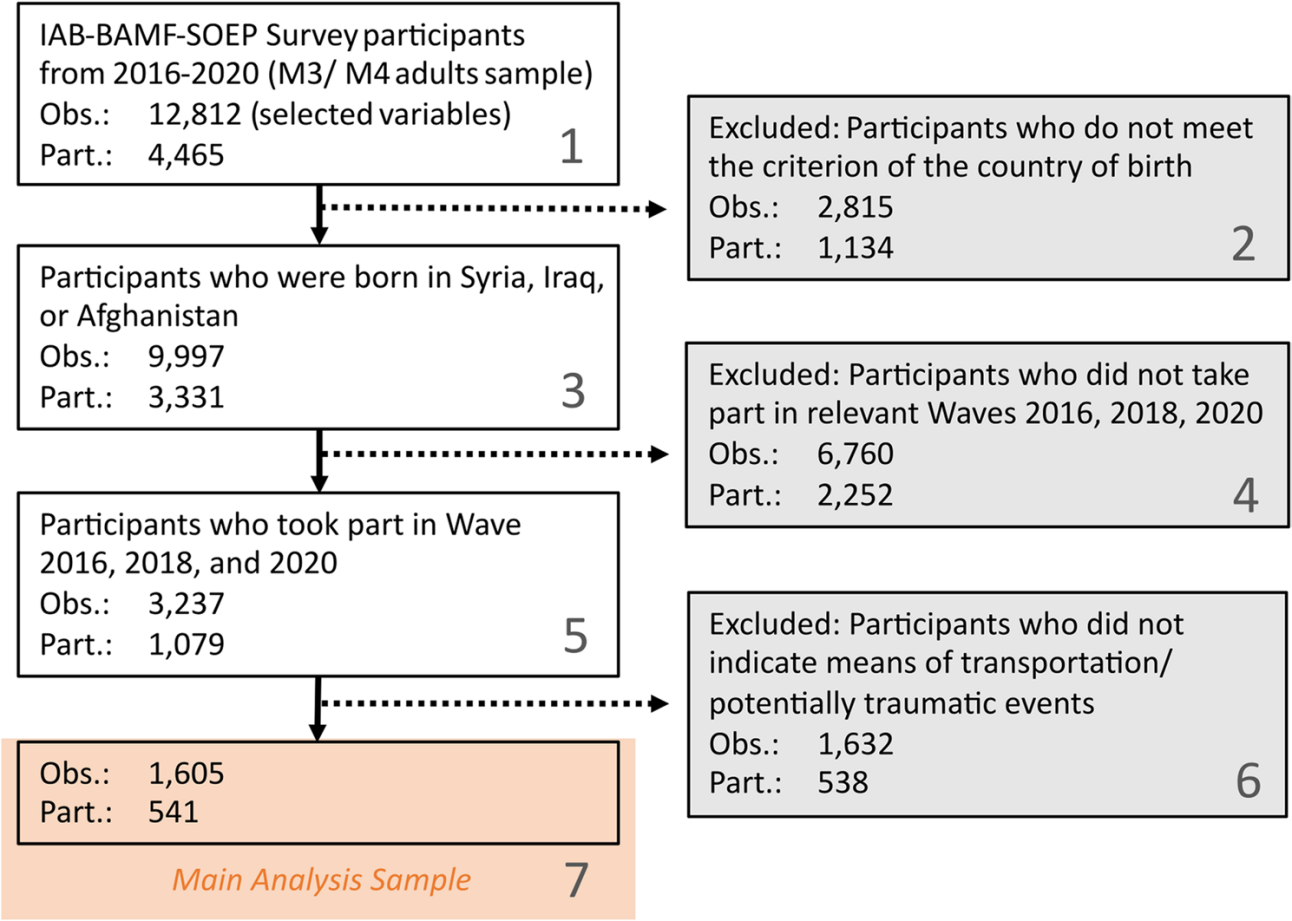
Participant flow chart. Obs. = observations, Part. = participants

The data set was filtered to include only refugees who were born in Middle Eastern countries, i.e. Syria, Iraq and Afghanistan. As questions on psychological distress were asked every two years, this study included data from the 2016, 2018 and 2020 waves. Participants with no available data in any of the three waves were excluded to maintain the accuracy of the analysis. This step was particularly important given the use of a causal mediation approach in later analyses, as missing data on mediator or outcome variables could lead to significant biases in the estimation of the indirect effect or the total effect (see, e.g., Imai et al. [44]). Figure 2 illustrates the participant flow chart.

### Measures

Time-invariant measures were collected in the first Wave in 2016, and time-variant measures in Wave 2016, 2018, and 2020. All variables used in the present study are listed in Table S1 in the Supplement with their original names as they are available in the SOEP-Core v38 dataset. The survey items were largely based on validated instruments from the SOEP-Core framework [41], including adaptations of existing scales to better reflect the migration context (e.g., wording adjustments, translation into multiple languages as described in"Data and ethical statement"section, and pretests) [42, 43, 45].

#### Indicators for peri-migration stressors (time-invariant)

##### Potentially traumatic events during migration

In the first wave of data collection in 2016, participants were asked whether they had experienced any of the following events during their forced migration to Germany (response format: 1 = Yes): (1) fraud or exploitation, (2) sexual harassment, (3) physical assault, (4) shipwreck, (5) robbery, (6) extortion, or (7) incarceration. If none of these applied, participants could select the option ‘None of these’. A sum score was created based on the number of events participants consented to having experienced in the distinct areas surveyed, with a value between 0 and 7. Higher values reflect a greater number of event types and thus a greater variety regarding PTEs experienced during migration.

##### Indicator for small boat crossing as a proxy for Mediterranean route use

A binary variable captured whether participants had used a small boat during their forced migration to Germany, based on reported modes of transportation including plane, ship, train, truck, car, bus, and walking. Participants who used a small boat were compared to those who did not (the latter served as a reference category; 0 = no/1 = yes). This provided a complementary indicator of a peri-migration stressor, likely stemming from the experience of crossing the Mediterranean by small boat.

#### Indicators for post-migration stress (time-variant)

##### Perceived discrimination

Participants were asked in a single item how often they had personally experienced discrimination in Germany over the past 24 months due to their origin. Responses were recorded on a 3-point scale (1 = ‘frequently’ to 3 = ‘rarely’) and recoded as a continuous variable, where higher values indicate more frequent perceptions of discrimination.

##### Migration-related worries

Respondents were asked to give a general assessment of the extent of their worries in areas consisting of (1) the asylum application, (2) their financial situation, (3) their health, (4) being unable to stay, and (5) the idea that they would never be able to return to their home country. Participants could indicate whether they had no, few, or major worries. Responses were quantified on a 3-point Likert scale (1 = ‘Yes, I worry a lot‘ to 3 = ‘I don’t worry at all’), and a mean score across all items was calculated, with higher values reflecting greater worries. Internal consistency was acceptable, with McDonald’s ω total values ranging from.62 to.68 across the three waves.

#### Indicator for mental health (time-variant)

Mental health was assessed using six items based on the Mental Component Score (MCS) from the original SF12v2 [46], a measurement of psychological distress that has proven to be cross-culturally applicable [47]. The items asked for the frequency of common depressive symptoms in the last 4 weeks, such as feeling exhausted, downhearted, or out of control due to emotional problems, on a 5-point scale (1 = ‘Very well’ to 5 = ‘Poor’). An overall mean score was calculated from all items. Higher scores indicate a greater frequency of these symptoms. Internal consistency was high, with McDonald’s ω total values ranging from 0.82 to 0.83 across the three waves.

#### Control variables (time-invariant and time-variant)

In addition to age and gender, the following control variables were included in the analyses and operationalized as follows:

##### Subjective assessment of relative income before migration (time-invariant)

Participants were asked to compare their net income from the time before they fled with that of other citizens of their home country based on 5 answer categories, ranging from “far below average” to “far above average”.

##### Support from relatives on arrival (time-invariant)

Participants were asked to indicate whether they had received support from relatives who were already in Germany when they arrived. A binary variable was created from the answers, with “No” as the reference category (0 = No/1 = Yes).

##### Housing situation (time-variant)

A binary variable was created to distinguish between private housing (0; reference category) and shared accommodation (1) based on participants’ current living situation. The latter mainly included shared accommodation in converted buildings (e.g. former office buildings and schools), (former) hotels and guest houses as well as temporary accommodation including containers or rapid assembly accommodation, halls and tents [48].

##### German language proficiency (time-variant)

Language proficiency was determined as an averaged sum value across three items that asked participants to self-assess their German language proficiency in the areas of speaking, reading and writing. The items could be answered on a 5-point Likert scale (1 = ‘Very well’ to 5 = ‘Not at all’). Internal consistency was high, with McDonald’s ω total values ranging from 0.93 to 0.94 across the three waves.

### Analysis strategy

All analyses were conducted using R 4.4.1 (R Core Team, 2024). To examine whether missing data on modes of transportation or PTEs during migration was systematically associated with participants’ age and gender, we conducted a logistic regression with missingness as the dependent variable. Power was estimated using the Monte Carlo approach for mediation models proposed by Schoemann et al. [49].

The causal mediation approach (see Imai et al. [44]) was employed to estimate the average direct (ADE) and average causal mediated effects (ACME) in longitudinal data as postulated in Fig. 1. ACME values were calculated by using the mediation R-package [50], which is multiplying the paths between the mediator (association between predictor and mediator) and outcome models (association between mediator and outcome as well as predictor and outcome). These models were estimated using linear mixed-effects models (LMMs) with only complete data regarding the model variables, incorporating random intercepts at the participant level to account for repeated measurements across the three survey waves (2016, 2018, 2020) and for data clustering within individuals. Quasi-Bayesian Monte Carlo simulations with 1,000 resamples were used to compute confidence intervals for the average direct and indirect effects, accounting for estimation uncertainty. The control variables were included in all models. Additionally, all models simultaneously included both peri-migration stressors (PTEs and small boat crossing) and both post-migration stress indicators (migration-related worries and perceived discrimination), either as predictors or as covariates, depending on the respective mediation pathway examined (see Fig. 1). As the event of shipwreck is also included among the PTEs, this adjustment allowed us to statistically control for potential confounding and thereby isolate the unique contribution of the small boat crossing as a broader contextual peri-migration stressor.

#### Robustness checks, post-hoc tests and sensitivity analyses

To examine significant differences in the indirect effects between gender (“Female”, “Male”) and age groups (“Under 39 years”, “40–60 years” in the first wave), mediation analyses were conducted separately for each group, estimating the ACME and ADE values. The age cut-off was pragmatically selected to approximate the sample median and ensure adequately sized subgroups for categorical moderation analyses within the mediation models. In addition, the threshold of 40 years aligns with theoretical definitions of the onset of midlife, which is commonly considered to begin around this age [51]. In line with the national refugee demographics [52], very few respondents in the IAB-BAMF-SOEP sample are over the age of 60. Consequently, we excluded this age group from the analysis. Group differences were determined by subtracting the respective ACME and ADE values between groups. A subsequent bootstrap procedure with 1,000 resamples was used to test the significance and stability of the group-specific effect differences, with confidence intervals calculated to account for variability.

In addition, we conducted sensitivity analyses on the significant mediation models that took into account the length of stay of participants in Germany, calculated as the number of months between arrival in Germany and the interview date for each wave. Furthermore, to test whether the results were influenced by specific legal admission pathways, we excluded participants who had received a humanitarian residence permit based on § 22 or § 23 of the German Residence Act, which are typically granted in resettlement or family reunification contexts.

Post-hoc tests included structural equation modeling (SEM) approaches that were employed to analyze differences in indirect effects between the three time points in the significant mediation models. Cross-sectional mediation models were calculated, whereby temporal covariances between mediators and outcomes (e.g. migration-related worries in 2016 with migration-related worries in 2018, or psychological distress in 2018 with psychological distress in 2020) were estimated to account for continuity and relationships between time points. Indirect effects were calculated using bootstrapping with 1,000 replicate samples. To examine whether the size of the indirect effects varied across survey waves, we conducted z-tests comparing ACME estimates and calculated bootstrapped confidence intervals for each comparison.

Furthermore, we performed two random intercept cross-lagged panel models (RI-CLPM) according to the approach of Hamaker et al. [53]. These models allow for disentangling within-person fluctuations from stable between-person differences across the three survey waves (2016, 2018, 2020). Given the exploratory aim and the potential contextual changes between survey periods, we estimated autoregressive and cross-lagged paths freely rather than constraining them to equality over time. This flexible specification enabled us to detect possible differences in dynamic processes between earlier and later intervals. Each model included either (1) migration-related worries or (2) perceived discrimination as time-varying predictors of psychological distress, with PTE exposure/small boat crossing as exogenous variables. Model fit was evaluated using χ², CFI, and SRMR.

## Results

### Participants and descriptive statistics

No significant associations were found between missingness and age (age_missing data_: *b* = 0.02, *SE* = 0.01, *p* =.061) or gender (gender_missing data_: *b* = −0.12, *SE* = 0.08, *p* =.150). Based on the inclusion criteria, the final sample consisted of *N* = 541 (*M*_age_ = 38.77, *SD*_age_ = 9.53). Details on the sampling strategy and exclusion criteria are provided in Fig. 2.

The final sample size was sufficient to achieve statistical power exceeding 90% for the tested mediation models. At the time of the first survey wave in 2016, 342 participants (63.2%) were under 39 years old, and 199 (36.8%) were between 40 and 60 years old. Most participants were born in Syria (*n* = 418; 77.3%), followed by Iraq (*n* = 72; 13.3%) and Afghanistan (*n* = 51; 9.4%), with predominantly male participants (roughly one in seven participants identified as female) which is representative of the composition of the Middle Eastern refugee group in Germany since 2016 according to official figures [52]. Participants arrived in Germany mainly in 2015 (*n* = 375; 69%), followed by 2014 (*n* = 118; 21%). A small proportion reported arriving in 2013 (*n* = 27; 4.9%) or in 2016 (*n* = 21; 3.8%). Among participants who reported exposure to at least one PTE during migration (*n* = 308; 57%), the average number of different PTE types experienced was slightly above two. The most frequently reported events were fraud/exploitation (*n* = 179; 58.1%) and incarceration (*n* = 125; 40.6%), while sexual harassment was reported least frequently (*n* = 9; 3%). A substantial portion of the sample (*n* = 233; 43%) indicated no exposure to any of the listed PTEs. Most participants estimated their pre-migration net income to be in the middle range compared to other citizens of their country of origin. Psychological distress decreased slightly, while German language skills showed a modest improvement over the course of participants’ stay. During their stay, participants increasingly moved from shared accommodation to private accommodation. Table 1 presents more detailed time-invariant participant characteristics assessed at wave 2016, whereas Table 2 displays time-varying characteristics measured across all three survey waves.

**Table 1:** Descriptive statistics of time-invariant participant characteristics in 2016 (*N* = 541)

Variable (time-invariant)	2016
Country of Birth	
Syria, *n* (%)	418 (77.3)
Iraq, *n* (%)	72 (13.3)
Afghanistan, *n* (%)	51 (9.4)
Gender	
Female, *n* (%)	76 (14.0)
Male, *n* (%)	465 (86.0)
PTE Sum, *n* (*SD*)	2.09 (1.3)
No PTEs, *n* (%)	233 (43.0)
*Relative Income Before Migration*	
Far Below Average, *n* (%)	56 (10.4)
Below Average, *n* (%)	91 (16.8)
Average, *n* (%)	246 (45.5)
Above Average, *n* (%)	95 (17.5)
Far Above Average, *n* (%)	53 (9.8)
Support from relatives on arrival, *n* (%)	97 (17.9)
*Modes of Transportation*	
Small Boat, *n* (%)	351 (64.9)
Plane, *n* (%)	169 (31.2)
Ship, *n* (%)	143 (26.4)
Train, *n* (%)	294 (54.3)
Truck, *n* (%)	109 (20.1)
Car, *n* (%)	287 (53.0)
Bus, *n* (%)	285 (52.6)
Walking, *n* (%)	381 (70.4)

PTE Sum = number of potentially traumatic events experienced during migration; Relative Income Before Migration = subjective assessment of relative income before migration. Percentages for modes of transportation are based on the total sample size; participants could report multiple modes

**Table 2:** Descriptive statistics of time-variant participant characteristics across survey waves (2016–2020, *N* = 541)

Variable (time-variant)	2016	2018	2020
Age, *M* (*SD*)	36.66 (9.29)	38.63 (9.53)	40.85 (9.54)
Psychological Distress, *M* (*SD*)	13.40 (4.77)	13.28 (4.62)	12.55 (4.21)
Perceived Discrimination, *M* (*SD*)	1.42 (0.60)	1.44 (0.58)	1.28 (0.50)
Migration-related Worries, *M* (*SD*)	1.70 (0.35)	1.74 (0.34)	1.60 (0.36)
Living Situation			
Communal, *n* (%)	155 (28.8)	56 (10.4)	40 (7.5)
Private, *n* (%)	383 (71.2)	480 (89.6)	497 (92.6)
German Language Proficiency, *M* (SD)	2.67 (0.91)	3.33 (0.82)	3.5 (0.8)

About two thirds of participants (*n* = 351; 64.9%) indicated that their migration involved a small boat crossing, with nearly one fifth of them (*n* = 68; 19.4%) also reporting a shipwreck experience. Among those who reported a small boat crossing, additional modes of transportation were frequently mentioned, including walking (*n* = 287; 81.8%), train (*n* = 245; 69.8%), bus (*n* = 219; 62.4%), car (*n* = 197; 56.1%), ship (*n* = 109; 31.0%), plane (*n* = 79; 22.5%), and truck (*n* = 75; 21.4%). Almost a quarter of this group (*n* = 124; 22.9%) reported no exposure to any of the listed PTEs during their migration. Among the one-third of participants who reported using modes of transportation other than small boats during their migration, walking was also most frequently reported (*n* = 92; 48.2%), followed by car (*n* = 90; 47.1%), airplane (*n* = 89; 46.6%), bus (*n* = 64; 33.5%), train (*n* = 48; 25.1%), truck (*n* = 34; 17.8%), and ship (*n* = 33; 17.3%). Of these, more than half (*n* = 109; 57%) reported they were not experiencing any PTEs. Complementary comparison analyses between participants who indicated a small boat crossing and those who did not revealed no significant differences in age, *t*(966.85) = 0.88, *p* =.381, gender, χ²(1) = 1.22, *p* =.269, or income level, *W* = 61,343, *p* =.865. While no significant differences were found in German language skills between the two groups in 2016, *t*(966.21) = − 1.26, *p* =.210, or in 2018, *t*(966.98) = − 1.47, *p* =.143, a small but statistically significant difference emerged in 2020, *t*(966.83) = − 2.06, *p* =.040, with participants reported a small boat crossing also indicated slightly higher language proficiency. Moreover, this group was significantly less likely to report social support from relatives on arrival, χ²(1) = 11.14, *p* <.001. In fact, nearly twice as many participants who used other modes of transportation than a small boat reported receiving support from relatives compared to those who indicated a small boat crossing. In terms of housing, participants who reported a small boat crossing were significantly more likely to reside in shared accommodation in 2016, χ²(1) = 13.10, *p* <.001, and 2020, χ²(1) = 9.15, *p* =.002, but not in 2018, χ²(1) = 0.74, *p* =.388, when compared with participants who used other modes of transportation. Furthermore, participants with small boat crossing exposure reported significantly higher levels of perceived discrimination, particularly in wave 2018, *t*(939.94) = − 2.46, *p* =.014, and wave 2020, *t*(886.60) = − 4.74, *p* <.001, and significantly higher number of distinct PTEs, *t*(496.95) = − 3.49, *p* <.001. No consistent differences were found in psychological distress or migration-related worries.

### Association between experiencing peri-migration stressors and perceiving post-migration stress (Hypothesis 1)

A higher number of reported PTEs was associated with more pronounced migration-related worries across multiple survey waves following arrival in the host country (β = 0.10, *CI* 95% [0.03, 0.12], *p* <.001; see mediator model in Table S2 in the Supplement). There was no significant association between PTEs and perceived discrimination upon or after arrival (see mediator model in Table S3 in the Supplement). Indicating a small boat crossing during migration was significantly related to increased discrimination experiences across the survey waves when compared to the use of other modes of transportation than a small boat (β = 0.11, *CI* 95% [0.11, 0.35], *p* <.001; see mediator model in Table S4 in the Supplement), while no effect was found in relation to subsequent migration-related worries (see mediator model in Table S5 in the Supplement).

### Mediation effects for post-migration stress regarding the relationship between the experience of peri-migration stressors and later psychological distress (Hypothesis 2)

**Fig. 3:**
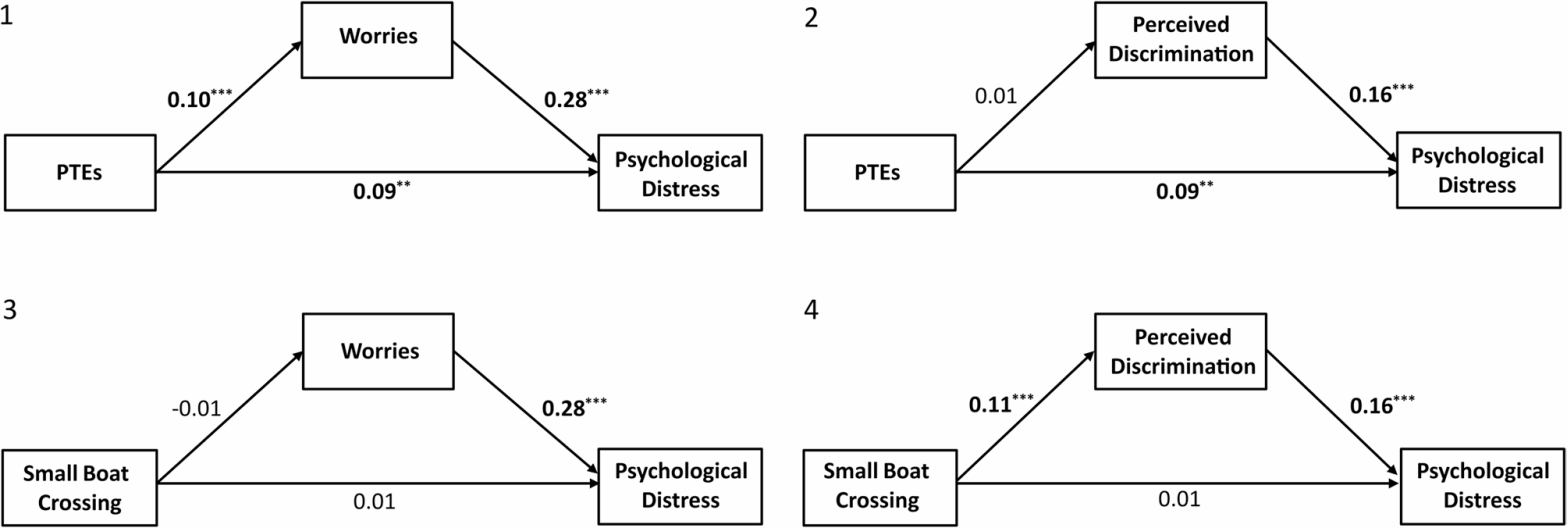
Path coefficients of the mediation models. All models were controlled for (1) age, (2) gender, (3) self-reported relative income before migration, (4) current living situation, (5) language proficiency in German, and (6) whether there was support from relatives when moving to Germany. Model 1: Predictor = PTE, mediator = migration-related worries, outcome = psychological distress. Model 2: Predictor = PTE, mediator = perceived discrimination, outcome = psychological distress. Model 3: Predictor = using vs. not using a small boat during migration, mediator = migration-related worries, outcome = psychological distress. Model 4: Predictor = using vs. not using a small boat during migration, mediator = perceived discrimination, outcome = psychological distress

The results for Model 1 showed a significant and positive indirect effect of PTEs on psychological distress via migration-related worries, with a mediation proportion of 26% (*ACME* = 0.02, *CI* 95% [0.01, 0.04], *p* <.001; see Table S6 in the Supplement). The direct effect was also significant and positive, suggesting that, taking the potential mediator into account, a higher number of PTE types experienced during migration is associated with higher psychological distress in the period after arrival in the host country (ADE = 0.07, *CI* 95% [0.03, 0.11], *p* <.001). In this model, the fixed effects explained 7.2% of the variance in migration-related worries and 17.9% in psychological distress (marginal *R*²), while the full models including random effects explained 26.4% and 32.3%, respectively (conditional *R*²). In Model 2, no significant indirect effect of PTEs on psychological distress via perceived discrimination was found (see Table S7 in the Supplement). The results for Model 3 provided no evidence of a significant indirect effect of small boat crossing and subsequent psychological distress via migration-related worries among participants. Additionally, no significant direct effect of small boat crossing on psychological distress was found (see Fig. 3 and Table S8 in the Supplement). However, perceived discrimination fully mediated the relationship between small boat crossing and psychological distress after migration in Model 4 (ACME = 0.05, CI 95% [0.02, 0.08], *p* <.001), with no significant direct effect observed in the model (see Fig. 3 and Table S9 in the Supplement). In Model 4, the marginal *R*² was 7.2% for perceived discrimination and 17.9% for psychological distress, while the conditional *R*² was 26.4% and 32.3%, respectively.

### Robustness checks, post-hoc tests and sensitivity analyses

The analysis of potential moderators revealed that the mediation pathway, linking small boat crossing to psychological distress through migration-related worries, significantly varied by age. Specifically, this pathway was more pronounced in participants aged 40 to 60 years, whereas it was weaker among younger participants (see Model 3 in Figure S1 in the Supplement). The difference test produced a bootstrapped confidence interval that excluded zero (*CI* 95% [−0.11, −0.01]). No other significant effects could be determined, indicating the stability of the main mediation findings across subgroups.

Sensitivity analyses that included length of stay in Germany (in months) as an additional covariate in Models 1 and 4 showed no substantial change in the strength, direction, or significance of the reported effects. A longer time since arrival was modestly associated with lower levels of migration-related worries (β = −0.11, *p* <.001), but showed no significant association with perceived discrimination or psychological distress.

Moreover, excluding participants (*N* = 34, 6.3%) who reported receiving a residence permit based on § 22 or § 23 of the German Residence Act yield that results remained unchanged with respect to effect size, direction, and statistical significance in Model 1 and Model 4, supporting the stability of the findings.

In addition, results of SEM to analyze differences in indirect effects between the three time points in the significant mediation models for Model 1 and Model 4 can be found in Tables S10– S12 in Supplement. These indicate that the indirect effects became stronger over time in both models: In Model 1, the indirect effect of PTEs on psychological distress via migration-related worries increased significantly from 2016 to 2020 (*Z* = − 2.45, *p* =.014) and from 2018 to 2020 (*Z* = − 2.50, *p* =.013). Similarly, in Model 4, the indirect effect of using a small boat in the Mediterranean on psychological distress via perceived discrimination was significantly larger in 2020 compared to both 2016 (*Z* = − 2.52, *p* =.012) and 2018 (*Z* = − 2.55, *p* =.011).

Furthermore, results of the two RI-CLPM that were estimated to additionally explore the temporal dynamics between PTEs/small boat crossing, (1) migration-related worries, and (2) perceived discrimination in relation to psychological distress across the three survey waves showed an excellent fit in the first model: χ²(5) = 5.61, *p* =.347, *CFI* = 0.98, and *SRMR* = 0.011. The latent correlation between migration-related worries and psychological distress between individuals was significant (*r* =.55, *p* <.001). At the participant level, autoregressive effects for migration-related worries were significant from wave 2016 to 2018 (β = 0.15, *p* =.011), but not from wave 2018 to 2020 (β = 0.11, *p* =.110). Cross-lagged effects of migration-related worries on subsequent psychological distress were not significant (β = 0.05–0.01, all *p*s > 0.25). The second model also showed excellent fit: χ²(5) = 3.57, *p* =.612, *CFI* = 0.98, *SRMR* = 0.011. The latent correlation between perceived discrimination and psychological distress was significant (*r* =.55, *p* <.001). Autoregressive effects for perceived discrimination were significant between wave 2016 and 2018 (β = 0.22, *p* <.001), but not from wave 2018 to 2020 (β = 0.04, *p* =.542). No significant cross-lag effects were found for perceived discrimination as a predictor of subsequent psychological distress (β = − 0.09 to 0.05, all *p*s > 0.09).

## Discussion

The aim of this study was to examine the association between stressors during and after migration and their potential influence on the mental health of refugees from the Middle East in Germany, based on a longitudinal data set. Results from the analyses indicate that experiencing peri-migration stressors may trigger the emergence of subsequent post-migration stress. Specifically, exposure to PTEs across a wider range of categories significantly predicted subsequent migration-related worries across three survey waves (Hypothesis 1). It is therefore possible that new potential stressors in the host country will become burdensome—not only because they can carry a high level of risk, but also because previous stress may have enabled them to take effect. This finding is consistent with previous studies demonstrating an association between trauma exposure before migration and persistent worries about safety, financial stability, and health after resettlement, and extends this evidence by showing that such associations also hold for stressors specifically experienced in the peri-migration phase [29, 34, 37, 54].

Notably, refugees who likely crossed the Mediterranean by boat reported significantly more discrimination after arriving in the host country than those who used other modes of transportation, while accounting for the cumulative effect of various PTEs, including the experience of a shipwreck. This effect underscores the importance of including alternative indicators for context-specific stressors relevant to the refugee group under study, such as the report of small boat crossings in terms of Middle Eastern (or African) refugees, as these have a unique risk context beyond PTEs. This is reinforced by the finding that around a quarter of those participants who reported having used a small boat along the migration route did not endorse any of the predefined PTE categories. Qualitative reports from previous studies suggest that the extreme violence encountered during migration—particularly in transit countries such as Libya—may lead individuals to cognitively reframe or emotionally detach from subsequent dangers, resulting in psychological numbing or fatalistic resignation [14].

However, contrary to our expectations, no significant association was found between PTEs and subsequent perceptions of discrimination. Furthermore, there were no differences in migration-related worries between refugees who indicated a small boat crossing and those who did not. This pattern may suggest that boat crossings are less relevant for the emergence of internalized persistent worries about future prospects, such as legal status, finances, or health. The different associations between peri-migration stressors and the two post-migration stress indicators examined may be due to the qualitatively distinct psychological nature of these constructs: While migration-related worries reflect enduring internal states of uncertainty and perceived lack of control over future life domains, perceived discrimination involves the interpretation of social cues and interpersonal interactions in the host context. Perceived discrimination—as a socially constructed stressor—can not only influence how previous experiences of powerlessness or imposed dependence, for example during the crossing [13], affect the interpretation of one’s current social position in the host country, but may also serve as a way of constructing and maintaining the psychological significance of past stressful situations in a context of structural inequality (see"Perceived discrimination and mental health following the small boat crossing"section). In contrast, migration-related worries may reflect a generalized sense of uncertainty about the future, potentially rooted in the cumulative experience of uncontrollable and multifaceted threats during migration. It is also conceivable that the greater worries of those who have experienced and suffered a larger number of PTEs result not only from existential uncertainties, but also from the fear that the hardships and risks associated with fleeing may ultimately have been in vain. Hence, their relevance for shaping how people interpret interpersonal behavior in resettlement settings may be different than for those who experienced small boat crossings. Thus, different peri-migration stressors may channel into post-migration stress via distinct psychological routes: boat crossings may prime individuals to detect exclusion in social contexts, while cumulative PTEs may erode confidence in personal agency and long-term security.

Results from causal mediation analyses indicate that migration-related worries have partially mediated the relationship between potentially traumatic experiences and psychological distress across time, since experiencing PTEs may additionally have a direct impact on mental health. On the other hand, fleeing by boat and subsequent psychological distress were fully mediated by perceived discrimination upon arrival (Hypothesis 2). Furthermore, post-hoc analyses suggest that the observed mediation effects may have intensified over time. While no significant lagged effects were identified in the sensitivity analyses, the pattern of results is consistent with the stress proliferation framework in that early stress exposures are stably associated with ongoing psychosocial burden and distress [33].

### Migration-related worries and mental health after experiencing potentially traumatic events during migration

Upon arrival in the host country, refugees face long-term challenges, including uncertainty about their future prospects and the difficulties of rebuilding their lives [55]. These challenges are compounded by post-migration stressors, such as institutional, social, and cultural barriers, which are part of a broader framework of social determinants of refugee mental health especially in the first years after their arrival [56]. While post-migration stressors refer to objective, external challenges (e.g., housing insecurity, financial constraints, or legal restrictions), this study focused on their subjective, psychological correlates, operationalized as migration-related worries and perceived discrimination. The mediation effect found in our study suggests that experiencing a greater variety of potentially traumatic events during migration may trigger a long-term stress response that is not adequately regulated [57]. This could leave those affected in a persistent state of ‘survival mode’– even after arrival in the host country– marked by an activation of stress responses, including high levels of vigilance, difficulty relaxing, and a focus on immediate threats or needs. The combination of prior trauma and new post-migration stressors could be perceived as an immediate threat to basic security, while there is a lack of resources to deal with them, also due to the refugee status. There may be a sense of lack of control, which can contribute to the emergence of migration-related worries in terms of a maladaptive coping strategy [58]. Consequently, this type of worry can act as a mediating mechanism linking the psychological effects of past trauma to current mental health outcomes in refugees.

### Perceived discrimination and mental health following the small boat crossing

The finding that refugees from the Middle East who likely crossed the Mediterranean by boat reported greater psychological distress via higher levels of perceived discrimination, compared to those who used other modes of transportation, aligns with the results of prior research with other marginalized groups. As argued by Matheson et al. [59], prior externally inflicted exposure to stressful life events which are not necessarily perceived as discriminatory initially—such as small boat crossings—can acquire socially ascribed meaning through ongoing encounters with unequal treatment, social marginalization, and institutional bias over time. For example, this can occur through disproportionate placement in collective housing, restrictive asylum procedures, limited access to health and social services, or persistent negative media portrayals of boat refugees [28]. Such conditions may retroactively alter the perceived meaning of the migration experience, transforming it into a node within a broader pattern of exclusion and inequality. In this way, a structurally embedded, politically charged migration pathway could become reinterpreted by refugees themselves as evidence of their devalued social position, reinforcing refugees’ own sense of stigmatization, contributing to a discrimination-related stress pathway and, in turn, to psychological distress. This interpretation is supported by the pattern observed in our data showing that the mediating role of perceived discrimination became more pronounced over time. It should be emphasized that this indirect association remained significant even after controlling for a wide range of demographic, socioeconomic, and contextual covariates. Additional group comparisons also showed that refugees who had indicated small boat crossings differed from others in several background characteristics: they reported a higher number of PTEs, lived more frequently in shared accommodation (in two out of three waves) and had received less social support upon arrival. These differences support the assumption of discriminatory meaning-making, as they reflect structural disadvantages and social positioning that not only shape the migration process, but also the way in which this process is subsequently interpreted and responded to by others. Such structural markers may not be directly attributable to stressors associated with migration, but they can reinforce feelings of exclusion and increase vulnerability to discrimination, thereby acting as contextual moderators or amplifiers within the broader stress proliferation model.

Hence, a key question concerns the psychological mechanisms through which the small boat crossing experience may contribute to long-term mental health outcomes. As outlined in"Stress related to migration before arrival"section, small boat crossings are marked by prolonged fear, loss of control, and dependency on third parties [13–15]. The adverse conditions characterizing small boat crossings may foster a generalized sense of distrust or perceived marginalization that particularly unfolds after arrival and serves as a psychosocial mechanism linking the experience of small boat crossings to subsequent discrimination perceptions, thereby contributing to the observed mediation pathway. This interpretation is supported by our findings showing that the association between small boat crossings and perceived discrimination was particularly pronounced in later survey waves (2018, 2020), and by sensitivity analyses indicating that the indirect effect via perceived discrimination even intensified over time.

### Strengths, limitations and future research

The strengths of this study lie in its longitudinal design and the large, representative sample of a specific marginalized group, which allows for a robust examination of the proposed relationships across time. Additionally, the broad consideration of broader PTEs as well as specific stressful experiences during migration and various post-migration stressors, along with pre- and post-migration control variables, provides a more comprehensive view of stress proliferation in the refugee context. However, there are several limitations that should be acknowledged: First, while the small boat variable likely captures crossings via the Mediterranean—given historical migration patterns during 2013–2016—it cannot be definitively concluded that all reported boat crossings occurred in this region. Second, it is necessary to consider that refugees’ socioeconomic status prior to migration may shape both the likelihood of undertaking a small boat crossing and their subsequent psychosocial vulnerability. This study accounted for participants’ self-reported relative income in their countries of origin, thereby partially capturing pre-existing social inequalities. While this reduces the risk of conflating economic disadvantage with the specific stress associated with boat crossings, it does not fully capture the structural conditions that may moderate or amplify stress proliferation processes. Furthermore, although small boat crossings are often perceived as a sign of economic disadvantage, existing evidence suggests that this mode of migration is typically associated with substantial financial costs, often exceeding those of alternative routes [60], thereby challenging its interpretation as a straightforward indicator of lower socioeconomic status. Third, while the study employs a causal mediation framework, the observational nature of the data precludes definitive causal inference. Although the temporal ordering of variables aligns with the theoretical model, the retrospective nature of certain measures and the discrete time points of data collection limit conclusions regarding the precise timing of processes (e.g., due to bias in measuring peri-migration stressors as for most participants of the present study, the recall period was approximately one year), and potential method effects arising from varying recall periods across constructs—such as longer periods for perceived discrimination versus shorter timeframes for psychological distress—should be considered when interpreting the results. Future research should also explore to what extent differences in recall periods may influence associations between post-migration stress indicators and mental health outcomes, as certain experiences (e.g., discrimination) may have long-lasting psychological effects that extend beyond the time of occurrence. Nevertheless, the repeated-measures design, theoretical rationale, and results of exploratory sensitivity analyses lend support to the plausibility of the proposed indirect pathways as reflecting relatively stable between-person associations in line with Stress Proliferation Theory, rather than evidence of time-lagged, dynamic within-person effects. Fourth, our operationalization of peri-migration stressors may be limited in its ability to reflect the multifaceted and often prolonged nature of migration trajectories—for example, regarding transit phases in third countries, where conditions such as prolonged stays in refugee camps, unstable shelter, or repeated exposure to threat may constitute additional, unmeasured stressors [61]. These co-occurring experiences may represent further mechanisms through which peri-migration stressors impact mental health. While our study offers important initial insights into these relationships, future research is needed to disentangle the complex pathways through which various peri-migration conditions exert their effects—ideally with more granular and time-sensitive data. Fifth, a trauma checklist to capture potentially traumatic events across various categories may not fully encompass the range of refugee experiences prior to arriving in the host country. The use of a sum score, which reflects the number of different trauma categories experienced, risks obscuring the distinct psychological effects of specific types of traumatic events. For example, sexual violence and robbery may have very different psychological consequences, which are not differentiated by the summed approach. However, previous research (e.g., Kolassa et al. [62]; Wilker et al. [63]) suggests that although more detailed assessments can theoretically provide additional insights, they often contribute little to predictive power and complicate the assessment process. Therefore, the summative approach represents a practical compromise that provides a balance between comprehensiveness and feasibility in large-scale studies. Compared to previous studies, our sample includes a relatively large proportion of participants (43%) who report no exposure to PTEs (see, e.g., Sigvardsdotter et al. [9]). The face-to-face nature of household interviews may have inhibited disclosure of particularly sensitive or stigmatized experiences—such as sexual violence or exploitation—due to concerns about privacy, social desirability, the presence of family members during the interview, or culturally mediated norms regarding the communication of trauma. Sixth, although the data stem from a nationally representative sampling frame of asylum seekers registered through regular procedures in Germany, the present sample is household-based and excludes younger refugee populations. As a result, adult individuals and family households are overrepresented, while younger and particularly vulnerable subgroups—such as unaccompanied minors—are underrepresented. This likely contributes to the higher average age in the present sample compared to other refugee studies in Germany, which often focus more on adolescents and young adults (e.g [7]). Accordingly, the generalizability of our findings to the broader population of forcibly displaced persons is limited.

### Conclusion

This study provides insights into the mental health outcomes of Middle Eastern refugees in Germany who arrived between 2013 and 2016 by examining the role of peri-migration and post-migration stress across time. The findings reveal that post-migration stress, such as migration-related worries or perceived discrimination, mediates the relationship of earlier peri-migration stress including traumatic experiences with psychological distress, aligning with the SPT. The proliferation of stress experiences that happened before individuals arrived in the host country suggests that there is need for targeted policy actions addressing both peri-migration stress experiences and ongoing challenges like perceived discrimination and economic insecurity in the host country.

## Supplementary Information

Supplementary Material 1.

## Data Availability

The data used in this study originate from the IAB-BAMF-SOEP Survey of Refugees and is publicly available in fully anonymized form (see https://www.doi.org/10.5684/soep.core.v38.1eu).

## Abbreviation

PTEs: Potentially traumatic events
