# Supplementary material for: Post-migration stress mediates associations between potentially traumatic peri-migration experiences and mental health among Middle Eastern refugees in Germany
Source: BMC Public Health. 2025 Jul 29;25:2582. doi: 10.1186/s12889-025-23660-w (PMC12306111; doi:10.1186/s12889-025-23660-w)

**##################################################################################
######## SUPPLEMENTARY MATERIAL ################################################
##################################################################################**

**Post-Migration Stress Mediates Associations Between Potentially Traumatic Peri-Migration Experiences and Mental Health among Middle Eastern Refugees in Germany**

Usama EL-Awad, Robert Eves, Justin Hachenberger, Kayvan Bozorgmehr, Theresa M. Entringer, Tobias Hecker, Oliver Razum, Odile Sauzet and Sakari Lemola

**TABLE OF CONTENTS**

**##################################################################################**

1. **Table S1:** Variables used in the present study, as available in the SOEP Core v38 dataset
2. **Table S2:** Mediator and Outcome Model (linear mixed-effects models) for Model 1: PTEs → Migration-related Worries → Psychological Distress
3. **Table S3:** Mediator and Outcome Model (linear mixed-effects models) for Model 2: PTEs → Perceived Discrimination → Psychological Distress
4. **Table S4:** Mediator and Outcome Model (linear mixed-effects models) for Model 3: Small Boat Crossing → Migration-related Worries → Psychological Distress
5. **Table S5:** Mediator and Outcome Model (linear mixed-effects models) for Model 4: Small Boat Crossing → Perceived Discrimination → Psychological Distress
6. **Table S6:** Causal mediation analysis for Model 1: PTEs → Migration-related Worries → Psychological Distress
7. **Table S7:** Causal mediation analysis for Model 2: PTEs → Perceived Discrimination → Psychological Distress
8. **Table S8:** Causal mediation analysis for Model 3: Small Boat Crossing → Migration-related Worries → Psychological Distress
9. **Table S9:** Causal mediation analysis for Model 4: Small Boat Crossing → Perceived Discrimination → Psychological Distress
10. **Table S10:** Post-hoc analysis: SEM parameter estimates for Model 1
11. **Table S11:** Post-hoc analysis: SEM parameter estimates for Model 4
12. **Table S12**: Post-hoc analysis: Bootstrapped difference tests for Indirect effects
13. **Figure S1:** Forest plot illustrating indirect, direct, and total effects from causal mediation analysis by age and gender groups

**Table S1**

*Variables used in the present study, as available in the SOEP Core v38 dataset*

| **Description** | **Wave 2016** | **Wave 2018** | **Wave 2020** |
| --- | --- | --- | --- |
| Person identifier | pid | pid | pid |
| Household identifier | hid_2016 | hid_2018 | hid_2020 |
| Survey year | syear | syear | syear |
| Year of birth | bgpbirthy | bipbirthy | bkpbirthy |
| Month of birth | bgpbirthm | bipbirthm | bkpbirthm |
| Gender | bgpsex | bipsex | bkpsex |
| Citizenship status | bgpr0101 | bip_518_q106 | bkp_328_q215 |
| Year of arrival in host country | bgpr3901 | bip_139_01_q106 | bkp_98_01_q215 |
| Living situation (private or communal) | bghr01 | bih_05_q116 | bkh_05_q205 |
| Perceived Discrimination | bgpr67 | bip_524_q106 | bkp_105_q216/ bkp_346_q215 |
| *Migration-related Worries:* |  |  |  |
| Worry asylum application | bgpr357 | bip_425_q106 | bkp_264_q215 |
| Worry financial situation | bgp14802 | bip_170_02 | bkp_168_02 |
| Worry about health | bgp14804 | bip_170_04 | bkp_168_04 |
| Worry about being unable to stay | bgpr358 | bip_426_q106 | bkp_265_q215 |
| Worry unable to return | bgpr359 | bip_427_q106 | bkp_266_q215 |
| *Psychological distress:* |  |  |  |
| SF-12 item 1 (MCS) | bgp10802 | bip_137_02 | bkp_126_02 |
| SF-12 item 2 (MCS) | bgp10803 | bip_137_03 | bkp_126_03 |
| SF-12 item 3 (MCS) | bgp10804 | bip_137_04 | bkp_126_04 |
| SF-12 item 4 (MCS) | bgp10808 | bip_137_08 | bkp_126_08 |
| SF-12 item 5 (MCS) | bgp10809 | bip_137_09 | bkp_126_09 |
| SF-12 item 6 (MCS) | bgp10810 | bip_137_10 | bkp_126_10 |
| *German language skills:* |  |  |  |
| Speaking German | bgpm_p_6101 | bip_95_q106 | bkp_58_q215 |
| Writing German | bgpm_p_6102 | bip_96_q106 | bkp_59_q215 |
| Reading German | bgpm_p_6103 | bip_97_q106 | bkp_60_q215 |
| Pre-flight SES | lr3041 |  |  |
| Support on arrival from relatives | lb1246 |  |  |
| *Potentially traumatic experiences during migration:* |  |  |  |
| Fraud/ exploitation | lr3122 |  |  |
| Sexual harassment | lr3123 |  |  |
| Physical assaults | lr3124 |  |  |
| Shipwreck | lr3125 |  |  |
| Robbery | lr3126 |  |  |
| Extortion | lr3127 |  |  |
| Incarceration | lr3128 |  |  |
| None of those | lr3129 |  |  |
| *Means of transportation:* |  |  |  |
| By car | lr3097 |  |  |
| By bus | lr3098 |  |  |
| By truck | lr3099 |  |  |
| By train | lr3100 |  |  |
| By plane | lr3101 |  |  |
| By ship | lr3102 |  |  |
| By boat | lr3103 |  |  |
| By foot | lr3104 |  |  |

**Table S2**

*Mediator and Outcome Model (linear mixed-effects models) for Model 1: PTEs → Migration-related Worries → Psychological Distress*

|  | **Mediator Model: Migration-related Worries** | | | | | **Outcome Model: Psychological Distress** | | | | |
| --- | --- | --- | --- | --- | --- | --- | --- | --- | --- | --- |
| *Predictors* | *Est.* | *Std. Beta* | *Conf. Int (95%)* | *Stand. CI* | *P-Value* | *Est.* | *Std. Beta* | *Conf. Int (95%)* | *Stand. CI* | *P-Value* |
| (Intercept) | -0.00 | 0.12 | -0.32 – 0.31 | -0.08 – 0.32 | 0.976 | -0.46 | -0.10 | -0.75 – -0.18 | -0.27 – 0.08 | **0.002** |
| PTE Total | 0.07 | 0.10 | 0.03 – 0.12 | 0.04 – 0.17 | **<0.001** | 0.06 | 0.09 | 0.03 – 0.10 | 0.04 – 0.14 | **0.001** |
| Income Below Average (vs. Far Below Average) | -0.21 | -0.22 | -0.45 – 0.02 | -0.45 – 0.02 | 0.074 | 0.13 | 0.13 | -0.08 – 0.34 | -0.08 – 0.34 | 0.214 |
| Income Average (vs. Far Below Average) | -0.15 | -0.15 | -0.36 – 0.05 | -0.36 – 0.05 | 0.145 | 0.00 | 0.00 | -0.18 – 0.18 | -0.18 – 0.18 | 0.986 |
| Income Above Average (vs. Far Below Average) | -0.13 | -0.13 | -0.37 – 0.10 | -0.37 – 0.11 | 0.272 | 0.05 | 0.05 | -0.15 – 0.26 | -0.15 – 0.26 | 0.608 |
| Income Far Above Average (vs. Far Below Average) | -0.04 | -0.04 | -0.30 – 0.23 | -0.31 – 0.24 | 0.794 | -0.03 | -0.03 | -0.27 – 0.21 | -0.27 – 0.21 | 0.790 |
| Age | 0.00 | 0.00 | -0.01 – 0.01 | -0.06 – 0.07 | 0.920 | 0.01 | 0.07 | 0.00 – 0.01 | 0.01 – 0.12 | **0.018** |
| Gender: Female (vs. Male) | 0.17 | 0.17 | 0.00 – 0.34 | 0.00 – 0.35 | **0.049** | 0.34 | 0.34 | 0.18 – 0.49 | 0.18 – 0.49 | **<0.001** |
| Living in a Communal Accommodation for Refugees (vs. in a Private Apartment or House) | 0.17 | 0.17 | 0.03 – 0.30 | 0.03 – 0.31 | **0.015** | 0.16 | 0.16 | 0.03 – 0.29 | 0.03 – 0.29 | **0.016** |
| Language Proficiency | -0.11 | -0.10 | -0.16 – -0.06 | -0.15 – -0.05 | **<0.001** | -0.06 | -0.06 | -0.12 – -0.01 | -0.11 – -0.01 | **0.016** |
| Relative Support: Yes (vs. No) | -0.19 | -0.19 | -0.35 – -0.03 | -0.36 – -0.03 | **0.020** | -0.04 | -0.04 | -0.18 – 0.10 | -0.18 – 0.10 | 0.600 |
| Small Boat Crossing | -0.01 | -0.00 | -0.13 – 0.12 | -0.06 – 0.06 | 0.935 | 0.03 | 0.01 | -0.08 – 0.14 | -0.04 – 0.07 | 0.620 |
| Perceived Discrimination | 0.13 | 0.13 | 0.08 – 0.17 | 0.08 – 0.17 | **<0.001** | 0.16 | 0.16 | 0.12 – 0.21 | 0.11 – 0.21 | **<0.001** |
| Migration-related Worries |  |  |  |  |  | 0.29 | 0.28 | 0.24 – 0.34 | 0.24 – 0.33 | **<0.001** |
| **Random Effects** | | | | | | | | | | |
| σ^2^ | 0.64 | | | | | 0.67 | | | | |
| τ_00_ | 0.26 | | | | | 0.14 | | | | |
| ICC | 0.29 | | | | | 0.18 | | | | |
| N | 541 | | | | | 541 | | | | |
| Observations | 1593 | | | | | 1593 | | | | |
| Marginal R^2^ / Conditional R^2^ | 0.062 / 0.333 | | | | | 0.179 / 0.323 | | | | |

*Note.* Models included random intercepts. Est. = Estimate; Std. = Standardized; Conf. Inf. = Confidence interval; PTE = number of potentially traumatic event types experienced during migration; Income Far/ Below/ Above Average = Subjective assessment of relative net income before migration; Relative Support = Whether participants received support from relatives when moving to Germany; Small Boat Crossing = Whether participants used a small boat as a mode of transportation during their migration.

**Table S3**

*Mediator and Outcome Model (*linear mixed-effects models*) for Model 2: PTEs →* Perceived Discrimination *→* Psychological Distress

|  | **Mediator Model: Perceived Discrimination** | | | | | **Outcome Model: Psychological Distress** | | | | |
| --- | --- | --- | --- | --- | --- | --- | --- | --- | --- | --- |
| *Predictors* | *Est.* | *Std. Beta* | *Conf. Int (95%)* | *Std, CI* | *P-Value* | *Est.* | *Std. Beta* | *Conf. Int (95%)* | *Std. CI* | *P-Value* |
| (Intercept) | 0.37 | -0.06 | 0.07 – 0.68 | -0.24 – 0.13 | **0.016** | -0.46 | -0.10 | -0.75 – -0.18 | -0.27 – 0.08 | **0.002** |
| PTE Total | 0.01 | 0.02 | -0.03 – 0.05 | -0.04 – 0.08 | 0.543 | 0.06 | 0.09 | 0.03 – 0.10 | 0.04 – 0.14 | **0.001** |
| Income Below Average (vs. Far Below Average) | -0.00 | -0.00 | -0.22 – 0.22 | -0.23 – 0.22 | 0.981 | 0.13 | 0.13 | -0.08 – 0.34 | -0.08 – 0.34 | 0.214 |
| Income Average (vs. Far Below Average) | 0.00 | 0.00 | -0.19 – 0.20 | -0.19 – 0.20 | 0.972 | 0.00 | 0.00 | -0.18 – 0.18 | -0.18 – 0.18 | 0.986 |
| Income Above Average (vs. Far Below Average) | 0.18 | 0.18 | -0.05 – 0.40 | -0.05 – 0.41 | 0.122 | 0.05 | 0.05 | -0.15 – 0.26 | -0.15 – 0.26 | 0.608 |
| Income Far Above Average (vs. Far Below Average) | 0.05 | 0.05 | -0.21 – 0.30 | -0.21 – 0.31 | 0.708 | -0.03 | -0.03 | -0.27 – 0.21 | -0.27 – 0.21 | 0.790 |
| Age | -0.01 | -0.14 | -0.02 – -0.01 | -0.20 – -0.08 | **<0.001** | 0.01 | 0.07 | 0.00 – 0.01 | 0.01 – 0.12 | **0.018** |
| Gender: Female (vs. Male) | 0.23 | 0.23 | 0.07 – 0.39 | 0.07 – 0.40 | **0.006** | 0.34 | 0.34 | 0.18 – 0.49 | 0.18 – 0.49 | **<0.001** |
| Living in a Communal Accommodation for Refugees (vs. in a Private Apartment or House) | -0.04 | -0.04 | -0.18 – 0.09 | -0.19 – 0.10 | 0.535 | 0.16 | 0.16 | 0.03 – 0.29 | 0.03 – 0.29 | **0.016** |
| Relative support: Yes (vs. No) | -0.04 | -0.04 | -0.19 – 0.11 | -0.20 – 0.11 | 0.592 | -0.04 | -0.04 | -0.18 – 0.10 | -0.18 – 0.10 | 0.600 |
| Language Proficiency | 0.05 | 0.05 | -0.00 – 0.11 | -0.00 – 0.10 | 0.053 | -0.06 | -0.06 | -0.12 – -0.01 | -0.11 – -0.01 | **0.016** |
| Small Boat Crossing | 0.23 | 0.11 | 0.11 – 0.35 | 0.06 – 0.17 | **<0.001** | 0.03 | 0.01 | -0.08 – 0.14 | -0.04 – 0.07 | 0.620 |
| Migration-related Worries | 0.14 | 0.14 | 0.09 – 0.19 | 0.09 – 0.19 | **<0.001** | 0.29 | 0.28 | 0.24 – 0.34 | 0.24 – 0.33 | **<0.001** |
| Perceived Discrimination |  |  |  |  |  | 0.16 | 0.16 | 0.12 – 0.21 | 0.11 – 0.21 | **<0.001** |
| **Random Effects** | | | | | | | | | | |
| σ^2^ | 0.72 | | | | | 0.67 | | | | |
| τ_00_ | 0.19 | | | | | 0.14 | | | | |
| ICC | 0.21 | | | | | 0.18 | | | | |
| N | 541 | | | | | 541 | | | | |
| Observations | 1593 | | | | | 1593 | | | | |
| Marginal R^2^ / Conditional R^2^ | 0.072 / 0.264 | | | | | 0.179 / 0.323 | | | | |

*Note.* Models included random intercepts. Est. = Estimate; Std. = Standardized; Conf. Inf. = Confidence interval; PTE = number of potentially traumatic event types experienced during migration; Income Far/ Below/ Above Average = Subjective assessment of relative net income before migration; Relative Support = Whether participants received support from relatives when moving to Germany; Small Boat Crossing = Whether participants used a small boat as a mode of transportation during their migration.

**Table S4**

*Mediator and Outcome Model (*linear mixed-effects models*) for Model 3: Small Boat Crossing → Migration-related Worries →* Psychological Distress

|  | **Mediator Model: Migration-related Worries** | | | | | **Outcome Model: Psychological Distress** | | | | |
| --- | --- | --- | --- | --- | --- | --- | --- | --- | --- | --- |
| *Predictors* | *Est.* | *Std. Beta* | *Conf. Int (95%)* | *Std. CI* | *P-Value* | *Est.* | *Std. Beta* | *Conf. Int (95%)* | *Std. CI* | *P-Value* |
| (Intercept) | -0.00 | 0.12 | -0.32 – 0.31 | -0.08 – 0.32 | 0.976 | -0.46 | -0.10 | -0.75 – -0.18 | -0.27 – 0.08 | **0.002** |
| Small Boat Crossing | -0.01 | -0.00 | -0.13 – 0.12 | -0.06 – 0.06 | 0.935 | 0.03 | 0.01 | -0.08 – 0.14 | -0.04 – 0.07 | 0.620 |
| Age | -0.21 | -0.22 | -0.45 – 0.02 | -0.45 – 0.02 | 0.074 | 0.13 | 0.13 | -0.08 – 0.34 | -0.08 – 0.34 | 0.214 |
| Income Below Average (vs. Far Below Average) | -0.15 | -0.15 | -0.36 – 0.05 | -0.36 – 0.05 | 0.145 | 0.00 | 0.00 | -0.18 – 0.18 | -0.18 – 0.18 | 0.986 |
| Income Average (vs. Far Below Average) | -0.13 | -0.13 | -0.37 – 0.10 | -0.37 – 0.11 | 0.272 | 0.05 | 0.05 | -0.15 – 0.26 | -0.15 – 0.26 | 0.608 |
| Income Above Average (vs. Far Below Average) | -0.04 | -0.04 | -0.30 – 0.23 | -0.31 – 0.24 | 0.794 | -0.03 | -0.03 | -0.27 – 0.21 | -0.27 – 0.21 | 0.790 |
| Income Far Above Average (vs. Far Below Average) | 0.00 | 0.00 | -0.01 – 0.01 | -0.06 – 0.07 | 0.920 | 0.01 | 0.07 | 0.00 – 0.01 | 0.01 – 0.12 | **0.018** |
| Gender: Female (vs. Male) | 0.17 | 0.17 | 0.00 – 0.34 | 0.00 – 0.35 | **0.049** | 0.34 | 0.34 | 0.18 – 0.49 | 0.18 – 0.49 | **<0.001** |
| Living in a Communal Accommodation for Refugees (vs. in a Private Apartment or House) | 0.17 | 0.17 | 0.03 – 0.30 | 0.03 – 0.31 | **0.015** | 0.16 | 0.16 | 0.03 – 0.29 | 0.03 – 0.29 | **0.016** |
| Relative Support: Yes (vs. No) | -0.19 | -0.19 | -0.35 – -0.03 | -0.36 – -0.03 | **0.020** | -0.04 | -0.04 | -0.18 – 0.10 | -0.18 – 0.10 | 0.600 |
| Language Proficiency | -0.11 | -0.10 | -0.16 – -0.06 | -0.15 – -0.05 | **<0.001** | -0.06 | -0.06 | -0.12 – -0.01 | -0.11 – -0.01 | **0.016** |
| PTE Total | 0.07 | 0.10 | 0.03 – 0.12 | 0.04 – 0.17 | **0.001** | 0.06 | 0.09 | 0.03 – 0.10 | 0.04 – 0.14 | **0.001** |
| Perceived Discrimination | 0.13 | 0.13 | 0.08 – 0.17 | 0.08 – 0.17 | **<0.001** | 0.16 | 0.16 | 0.12 – 0.21 | 0.11 – 0.21 | **<0.001** |
| Migration-related Worries |  |  |  |  |  | 0.29 | 0.28 | 0.24 – 0.34 | 0.24 – 0.33 | **<0.001** |
| **Random Effects** | | | | | | | | | | |
| σ^2^ | 0.64 | | | | | 0.67 | | | | |
| τ_00_ | 0.26 | | | | | 0.14 | | | | |
| ICC | 0.29 | | | | | 0.18 | | | | |
| N | 541 | | | | | 541 | | | | |
| Observations | 1593 | | | | | 1593 | | | | |
| Marginal R^2^ / Conditional R^2^ | 0.062 / 0.333 | | | | | 0.179 / 0.323 | | | | |

*Note.* Models included random intercepts. Est. = Estimate; Std. = Standardized; Conf. Inf. = Confidence interval; PTE = number of potentially traumatic event types experienced during migration; Income Far/ Below/ Above Average = Subjective assessment of relative net income before migration; Relative Support = Whether participants received support from relatives when moving to Germany; Small Boat Crossing = Whether participants used a small boat as a mode of transportation during their migration.

**Table S5**

*Mediator and Outcome Model (*linear mixed-effects models*) for Model 4: Small Boat Crossing → Perceived Discrimination →* Psychological Distress

|  | **Mediator Model: Perceived Discrimination** | | | | | **Outcome Model: Psychological Distress** | | | | |
| --- | --- | --- | --- | --- | --- | --- | --- | --- | --- | --- |
| *Predictors* | *Est.* | *Std. Beta* | *Conf. Int (95%)* | *Std. CI* | *P-Value* | *Est.* | *Std. Beta* | *Conf. Int (95%)* | *Std. CI* | *P-Value* |
| (Intercept) | 0.37 | -0.06 | 0.07 – 0.68 | -0.24 – 0.13 | **0.016** | -0.46 | -0.10 | -0.75 – -0.18 | -0.27 – 0.08 | **0.002** |
| Small Boat Crossing | 0.23 | 0.11 | 0.11 – 0.35 | 0.06 – 0.17 | **<0.001** | 0.03 | 0.01 | -0.08 – 0.14 | -0.04 – 0.07 | 0.620 |
| Age | -0.00 | -0.00 | -0.22 – 0.22 | -0.23 – 0.22 | 0.981 | 0.13 | 0.13 | -0.08 – 0.34 | -0.08 – 0.34 | 0.214 |
| Income Below Average (vs. Far Below Average) | 0.00 | 0.00 | -0.19 – 0.20 | -0.19 – 0.20 | 0.972 | 0.00 | 0.00 | -0.18 – 0.18 | -0.18 – 0.18 | 0.986 |
| Income Average (vs. Far Below Average) | 0.18 | 0.18 | -0.05 – 0.40 | -0.05 – 0.41 | 0.122 | 0.05 | 0.05 | -0.15 – 0.26 | -0.15 – 0.26 | 0.608 |
| Income Above Average (vs. Far Below Average) | 0.05 | 0.05 | -0.21 – 0.30 | -0.21 – 0.31 | 0.708 | -0.03 | -0.03 | -0.27 – 0.21 | -0.27 – 0.21 | 0.790 |
| Income Far Above Average (vs. Far Below Average) | -0.01 | -0.14 | -0.02 – -0.01 | -0.20 – -0.08 | **<0.001** | 0.01 | 0.07 | 0.00 – 0.01 | 0.01 – 0.12 | **0.018** |
| Gender: Female (vs. Male) | 0.23 | 0.23 | 0.07 – 0.39 | 0.07 – 0.40 | **0.006** | 0.34 | 0.34 | 0.18 – 0.49 | 0.18 – 0.49 | **<0.001** |
| Living in a Communal Accommodation for Refugees (vs. in a Private Apartment or House) | -0.04 | -0.04 | -0.18 – 0.09 | -0.19 – 0.10 | 0.535 | 0.16 | 0.16 | 0.03 – 0.29 | 0.03 – 0.29 | **0.016** |
| Relative Support: Yes (vs. No) | -0.04 | -0.04 | -0.19 – 0.11 | -0.20 – 0.11 | 0.592 | -0.04 | -0.04 | -0.18 – 0.10 | -0.18 – 0.10 | 0.600 |
| Language Proficiency | 0.05 | 0.05 | -0.00 – 0.11 | -0.00 – 0.10 | 0.053 | -0.06 | -0.06 | -0.12 – -0.01 | -0.11 – -0.01 | **0.016** |
| PTE Total | 0.01 | 0.02 | -0.03 – 0.05 | -0.04 – 0.08 | 0.543 | 0.06 | 0.09 | 0.03 – 0.10 | 0.04 – 0.14 | **0.001** |
| Migration-related Worries | 0.14 | 0.14 | 0.09 – 0.19 | 0.09 – 0.19 | **<0.001** | 0.29 | 0.28 | 0.24 – 0.34 | 0.24 – 0.33 | **<0.001** |
| Perceived Discrimination |  |  |  |  |  | 0.16 | 0.16 | 0.12 – 0.21 | 0.11 – 0.21 | **<0.001** |
| **Random Effects** | | | | | | | | | | |
| σ^2^ | 0.72 | | | | | 0.67 | | | | |
| τ_00_ | 0.19 | | | | | 0.14 | | | | |
| ICC | 0.21 | | | | | 0.18 | | | | |
| N | 541 | | | | | 541 | | | | |
| Observations | 1593 | | | | | 1593 | | | | |
| Marginal R^2^ / Conditional R^2^ | 0.072 / 0.264 | | | | | 0.179 / 0.323 | | | | |

*Note.* Models included random intercepts. Est. = Estimate; Std. = Standardized; Conf. Inf. = Confidence interval; PTE = number of potentially traumatic event types experienced during migration; Income Far/ Below/ Above Average = Subjective assessment of relative net income before migration; Relative Support = Whether participants received support from relatives when moving to Germany; Small Boat Crossing = Whether participants used a small boat as a mode of transportation during their migration.

**Table S6**

*Causal mediation analysis for Model 1: PTEs → Migration-related Worries →* Psychological Distress

|  | Estimate | 95% CI Lower | 95% CI Upper | P-Value |
| --- | --- | --- | --- | --- |
| ACME | 0.02 | 0.01 | 0.04 | **< 0.001** |
| ADE | 0.07 | 0.03 | 0.11 | **< 0.001** |
| Total Effect | 0.09 | 0.05 | 0.13 | **< 0.001** |
| Prop. Mediated | 0.26 | 0.12 | 0.47 | **< 0.001** |

*Note.* ACME = Average causal mediation effect; ADE = average direct effect.

**Table S7**

*Causal mediation analysis for Model 2: PTEs → Perceived Discrimination →* Psychological Distress

|  | Estimate | 95% CI Lower | 95% CI Upper | P-Value |
| --- | --- | --- | --- | --- |
| ACME | 0.01 | -0.01 | 0.02 | 0.136 |
| ADE | 0.09 | 0.05 | 0.13 | **0.002** |
| Total Effect | 0.09 | 0.05 | 0.13 | **< 0.001** |
| Prop. Mediated | 0.06 | -0.02 | 0.18 | 0.136 |

*Note.* ACME = Average causal mediation effect; ADE = average direct effect.

**Table S8**

*Causal mediation analysis for Model 3: Small Boat Crossing → Migration-related Worries →* Psychological Distress

|  | Estimate | 95% CI Lower | 95% CI Upper | P-Value |
| --- | --- | --- | --- | --- |
| ACME | 0.01 | -0.01 | 0.04 | 0.968 |
| ADE | 0.09 | -0.01 | 0.18 | 0.058 |
| Total Effect | 0.10 | -0.01 | 0.19 | 0.062 |
| Prop. Mediated | 0.02 | -0.09 | 0.73 | 0.926 |

*Note.* ACME = Average causal mediation effect; ADE = average direct effect.

**Table S9**

*Causal mediation analysis for Model 4: Small Boat Crossing → Perceived Discrimination →* Psychological Distress

|  | Estimate | 95% CI Lower | 95% CI Upper | P-Value |
| --- | --- | --- | --- | --- |
| ACME | 0.05 | 0.02 | 0.08 | **< 0.001** |
| ADE | 0.09 | -0.07 | 0.17 | 0.450 |
| Total Effect | 0.10 | -0.02 | 0.22 | 0.130 |
| Prop. Mediated | 0.45 | 0.01 | 0.84 | **0.046** |

*Note.* ACME = Average causal mediation effect; ADE = average direct effect.

**Table S10**

*Post-hoc analysis: SEM parameter estimates for Model 1*

| **Path** | ***B (SE)*** | ***Z*** | ***p*** | **β** | **95% *CI* Boot** |
| --- | --- | --- | --- | --- | --- |
| **Migration-related Worries (2016)** |  |  |  |  |  |
| PTE Total → Migration-related Worries (2016) | 0.114 (0.025) | 4.605 | <.001 | 0.166 | [0.066, 0.163] |
| **Psychological Distress (2016)** |  |  |  |  |  |
| Migration-related Worries (2016) → Psychological Distress (2016) | 0.327 (0.033) | 9.852 | <.001 | 0.301 | [0.262, 0.392] |
| PTE Total → Psychological Distress (2016) | 0.073 (0.026) | 2.772 | .006 | 0.098 | [0.021, 0.125] |
| **Migration-related Worries (2018)** |  |  |  |  |  |
| PTE Total → Migration-related Worries (2018) | 0.063 (0.027) | 2.372 | .018 | 0.088 | [0.011, 0.115] |
| **Psychological Distress (2018)** |  |  |  |  |  |
| Migration-related Worries (2018) → Psychological Distress (2018) | 0.385 (0.028) | 13.715 | <.001 | 0.388 | [0.330, 0.441] |
| PTE Total → Psychological Distress (2018) | 0.052 (0.024) | 2.143 | .032 | 0.073 | [0.004, 0.099] |
| **Migration-related Worries (2020)** |  |  |  |  |  |
| PTE Total → Migration-related Worries (2020) | 0.070 (0.024) | 2.860 | .004 | 0.106 | [0.022, 0.117] |
| **Psychological Distress (2020)** |  |  |  |  |  |
| Migration-related Worries (2020) → Psychological Distress (2020) | 0.362 (0.031) | 11.857 | <.001 | 0.353 | [0.302, 0.422] |
| PTE Total → Psychological Distress (2020) | 0.031 (0.023) | 1.324 | .185 | 0.045 | [-0.015, 0.076] |

*Note*. *N* = 969. CI Boot = bootstrapped 95% confidence intervals (1,000 resamples). SEM was conducted using Maximum Likelihood Estimation (ML) and Full Information-Maximum Likelihood (FIML) to account for missing data​. Fit indices: *CFI* = 0.902, *SRMR* = 0.084. Covariances: Migration-related worries (2016) with Migration-related worries (2018): *r* = 0.275, *p* < .001; Migration-related worries (2018) with Migration-related worries (2020): *r* = 0.288, *p* < .001; Psychological Distress (2016) with Psychological Distress (2018): *r* = 0.200, *p* < .001; Psychological Distress (2018) with Psychological Distress (2020): *r* = 0.179, *p* < .001.

**Table S11**

*Post-hoc analysis: SEM parameter estimates for Model 4*

| **Path** | ***B (SE)*** | ***Z*** | ***p*** | **β** | **95% *CI* Boot** |
| --- | --- | --- | --- | --- | --- |
| **Migration-related Worries (2016)** |  |  |  |  |  |
| PTE Total → Migration-related Worries (2016) | 0.114 (0.025) | 4.605 | <.001 | 0.166 | [0.066, 0.163] |
| **Psychological Distress (2016)** |  |  |  |  |  |
| Migration-related Worries (2016) → Psychological Distress (2016) | 0.327 (0.033) | 9.852 | <.001 | 0.301 | [0.262, 0.392] |
| PTE Total → Psychological Distress (2016) | 0.073 (0.026) | 2.772 | .006 | 0.098 | [0.021, 0.125] |
| **Migration-related Worries (2018)** |  |  |  |  |  |
| PTE Total → Migration-related Worries (2018) | 0.063 (0.027) | 2.372 | .018 | 0.088 | [0.011, 0.115] |
| **Psychological Distress (2018)** |  |  |  |  |  |
| Migration-related Worries (2018) → Psychological Distress (2018) | 0.385 (0.028) | 13.715 | <.001 | 0.388 | [0.330, 0.441] |
| PTE Total → Psychological Distress (2018) | 0.052 (0.024) | 2.143 | .032 | 0.073 | [0.004, 0.099] |
| **Migration-related Worries (2020)** |  |  |  |  |  |
| PTE Total → Migration-related Worries (2020) | 0.070 (0.024) | 2.860 | .004 | 0.106 | [0.022, 0.117] |
| **Psychological Distress (2020)** |  |  |  |  |  |
| Migration-related Worries (2020) → Psychological Distress (2020) | 0.362 (0.031) | 11.857 | <.001 | 0.353 | [0.302, 0.422] |
| PTE Total → Psychological Distress (2020) | 0.031 (0.023) | 1.324 | .185 | 0.045 | [-0.015, 0.076] |

*Note*. *N* = 969. CI Boot = bootstrapped 95% confidence intervals (1,000 resamples). SEM was conducted using Maximum Likelihood Estimation (ML) and Full Information-Maximum Likelihood (FIML) to account for missing data​. *CFI* = 0.862, *SRMR* = 0.058. Covariances: Perceived discrimination (2016) with Perceived discrimination (2018): *r* = 0.319, *p* < .001; Perceived discrimination (2018) with Perceived discrimination (2020): *r* = 0.161, *p* < .001; Psychological Distress (2016) with Psychological Distress (2018): *r* = 0.266, *p* < .001; Psychological Distress (2018) with Psychological Distress (2020): *r* = 0.215, *p* < .001.

**Table S12**

*Post-hoc analysis: Bootstrapped difference tests for Indirect effects*

| **Effect** | ***B (SE)*** | ***Z*** | ***p*** | **95% *CI* Boot** |
| --- | --- | --- | --- | --- |
| **Modell 1: PTE Total** |  |  |  |  |
| Indirect (2016) | 0.037 (0.009) | 4.216 | **<.001** | [0.020, 0.055] |
| Indirect (2018) | 0.024 (0.010) | 2.344 | **.019** | [0.004, 0.045] |
| Indirect (2020) | 0.025 (0.009) | 2.788 | **.005** | [0.007, 0.043] |
| Difference (2016 vs. 2018) | -0.001 (0.019) | -0.040 | .968 | [-0.038, 0.037] |
| Difference (2018 vs. 2020) | -0.055 (0.022) | -2.495 | .013 | [-0.098, -0.013] |
| Difference (2016 vs. 2020) | -0.055 (0.023) | -2.446 | .014 | [-0.100, -0.013] |
| **Modell 4: Small Boat Crossing** |  |  |  |  |
| Indirect (2016) | 0.031 (0.014) | 2.267 | .023 | [0.004, 0.058] |
| Indirect (2018) | 0.032 (0.012) | 2.553 | .011 | [0.007, 0.056] |
| Indirect (2020) | 0.086 (0.020) | 4.234 | <.001 | [0.046, 0.126] |
| Difference (2016 vs. 2018) | -0.001 (0.018) | -0.042 | .967 | [-0.036, 0.035] |
| Difference (2018 vs. 2020) | -0.054 (0.021) | -2.548 | **.011** | [-0.095, -0.013] |
| Difference (2016 vs. 2020) | -0.055 (0.022) | -2.518 | **.012** | [-0.098, -0.013] |

*Note. CI* Boot = bootstrapped 95% confidence intervals (1,000 resamples).

**Figure S1**

*Forest plot illustrating indirect, direct, and total effects from causal mediation analysis by gender and age groups*


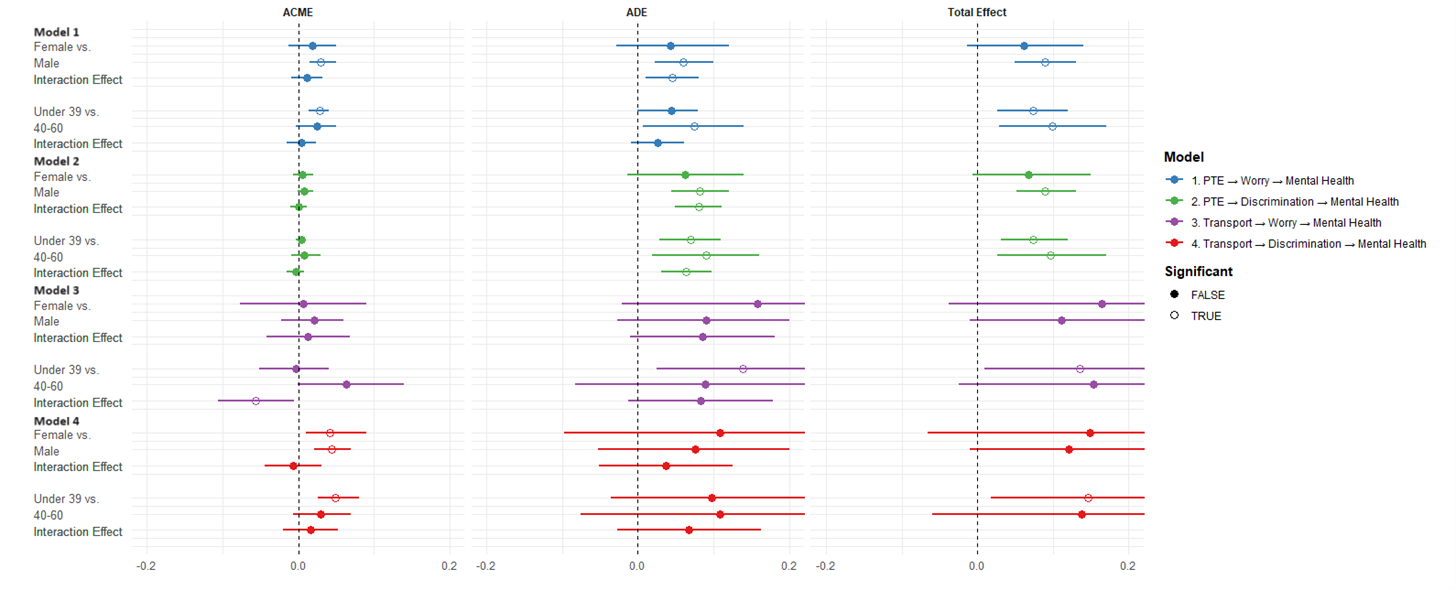

Supplement: Supplementary file 1 — Supplementary Material 1. [file 12889_2025_23660_MOESM1_ESM.docx]
